# 

**Published:** 1861-04

**Authors:** 


☞ The Philadelphia Reporter of 23rd February, accusing us of incorrigibility, did not reach us at the proper time. It however, did come in company with the succeeding issue of that Journal, after our March number had gone to press. We would only say to the Recorder, that it will always find us "incorrigible," when called upon by National assemblies, Philadelphia editors, or those of smaller pretentions, to indorse detestable principles; and my opinion is, that when objection to such cannot be answered by their advocates, justice, honesty and a good disposition require that they be given up without a *murmur.*

---

☞ Among the late additions to the Museum of the Atlanta Medical College, we find a pathological specimen of *Elephantiasis*, received by Dr. Brown, from Dr. D. S. Brandon, of Thomasville, Ga. From the short account given by Dr. Brandon, we learn that the penis and scrotum of the subject from which the specimen was taken, had been affected with this disease for about six years. The prepuce, which is the specimen sent, became so enlarged that micturition was interfered with, particularly under the appliances thought necessary in the treatment. Under these circumstances, excision became necessary. The portion excised measures about three and a half inches in length, and nearly two and a half inches thick. We hope to hear more of the case.

**List of Payments.**

Drs. W. C. Patterson, Ala., $5; H. A. Shaw, S. C., $10; R. T. Graves, Va., $6; G. H. Leitch, Va., $4; P. Timberlake, Ga. $6; P. E. Jennings, Ga., $6; O. H. Paul, Ga., $10; B. F. C. Bonnor, $3; J. W. Collier, Ga., $2; L. J. Dupree, Ga., $6; H. A. Venable, Va., $2; W. A. Watson, Ga., $4.50; C. S. Pittman, Ala., $3; A. Clements, Ga., $3; Thos. F. Duffey, N. C., $6; A. B. Spruill, Al., $5; D. W. Momand, Ga., $2; W. Hobbs, Ga., $2; J. B. Watkins, Ga., $3; G. B. Thomson, Tenn., $16.50; A. T. Henry, Ga. $3.

### TO CORRESPONDENTS.

By a recent change in the management of the Journal, its whole business affairs will hereafter come under the supervision of the Editor, and it is therefore requested that ALL Communications, whether relating to the Editorial, Subscription, Advertising or Financial Departments, should be addressed to J. G. WESTMORELAND, or Editor of Medical and Surgical Journal, Atlanta, Ga.

